# Reactive Lymphoid Hyperplasia of the Liver Incidentally Found in a 55-Year-Old Woman with a History of Ulcerative Colitis

**DOI:** 10.1155/2024/9863411

**Published:** 2024-02-22

**Authors:** Noritoshi Mizuta, Takuya Kikuchi, Shunsuke Kusano, Nobuya Sano

**Affiliations:** ^1^Department of Surgery, Akashi Medical Center, Akashi, Hyogo, Japan; ^2^Department of Diagnostic Pathology, Akashi Medical Center, Akashi, Hyogo, Japan

## Abstract

Reactive lymphoid hyperplasia (RLH) is a benign disease, rarely occurring in the liver. Reactive immune phenomenon has been reported in association with its occurrence, but the true pathogenesis is unknown. No case was reported in association with inflammatory bowel disease. We report a case of RLH of the liver in a patient with ulcerative colitis (UC). A 55-year-old woman with UC went to the outpatient clinic with abdominal pain, and antibiotics were prescribed with diagnosis of acute appendicitis. Imaging study detected a mass in the liver but ruled out appendicitis. She was referred to our hospital for further examination after pain improving. A 12 mm hypoechoic mass was detected in the liver on ultrasonography. There were no typical malignant findings on computed tomography and magnetic resonance imaging. Regular image follow-up was recommended, but the patient strongly requested surgery because of family history of malignant disease. Laparoscopic partial hepatectomy was performed. Histopathological findings revealed a conglomerate hyperplasia of lymphoid follicles with germinal centers. Infiltrating lymphocytes were non-neoplastic. Final diagnosis was RLH of the liver. UC is chronic inflammatory bowel disease and may be related to RLH, but there is no clear explanation at this point. This is the first known reported case of RLH of the liver in a patient with UC. But the relationship between the RLH and UC remains uncertain. Further investigation and case accumulation are necessary.

## 1. Introduction

Reactive lymphoid hyperplasia (RLH), also known as pseudolymphoma or nodular lymphoid lesion [1, 2], is a benign disease that occurs in various organs, such as the lungs, the orbits, the skin, and the gastrointestinal tract, however rarely occurring in the liver [1]. RLH is characterized by the proliferation of non-neoplastic polyclonal lymphocytes that form follicles with reactive germinal centers [2]. The first reported case of the RLH of the liver was by Snover et al. in 1981 [3]. The true pathogenesis is unknown, but its occurrence is thought to be associated with reactive immune phenomenon to various immune stimulators, such as chronic viral hepatitis, autoimmune disease, and malignant tumor [2, 3]. No cases occurring in patients with inflammatory bowel disease have been reported to date. We present a case of RLH of the liver in a patient with ulcerative colitis (UC).

## 2. Case Presentation

A 55-year-old woman with a seventeen-year history of UC presented with sudden but manageable localized right lower abdominal pain and went to an outpatient clinic. Oral sitafloxacin hydrate was prescribed with a presumed diagnosis of acute appendicitis. The pain did not improve, and the patient presented again to the emergency. Ultrasonography (US) and abdominal computed tomography (CT) ruled out appendicitis, but a mass was detected in the liver. The same antibiotic was continued and the crystalloid fluid was instilled, and the pain improved. The patient was then referred because of concern about exacerbation of UC and the malignant potential of the liver mass. No abdominal pain was noted.

**Figure 1:**
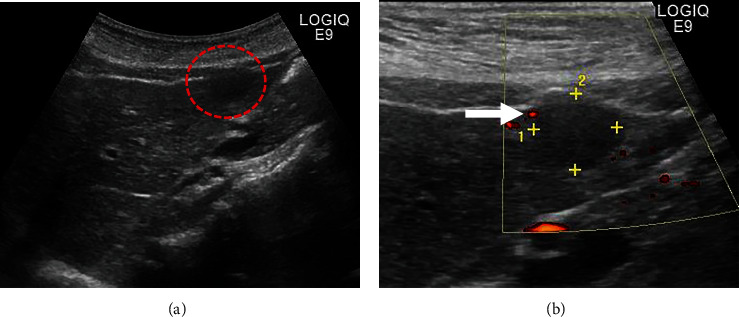
US findings. (a) On US, a low echoic mass was pointed out in segment 3 of the liver (red dotted area). (b) Blood flow signal was observed around the mass (white arrow).

**Figure 2:**
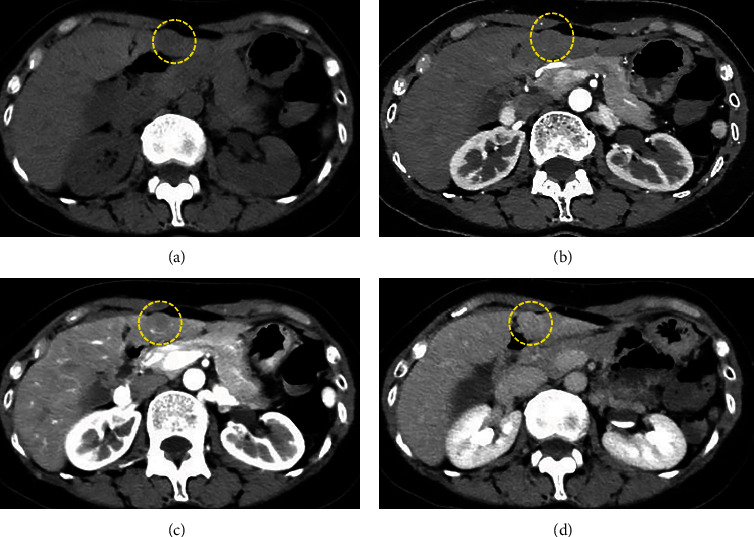
CT findings. (a) On plain CT, the mass was detected as low-density lesion. (b) The mass was not enhanced on contrast-enhanced CT in arterial phase. (c) The mass was slightly enhanced in portal phase. (d) The mass was detected as low-density lesion in delayed phase (yellow-dotted area).

**Figure 3:**
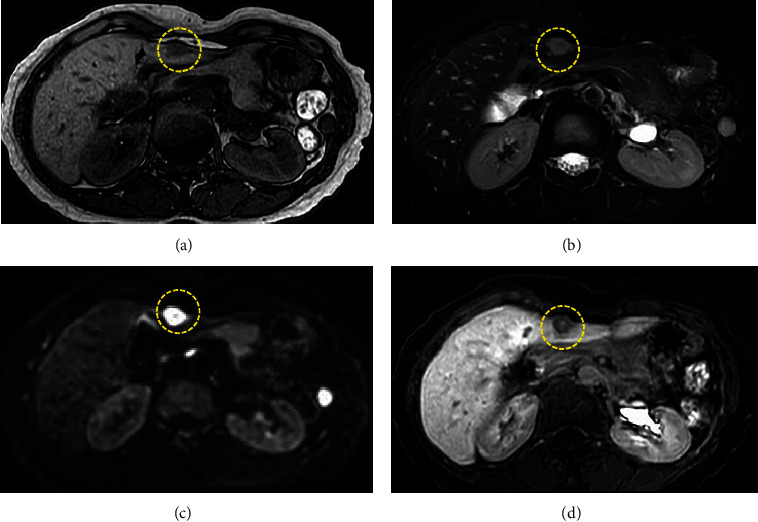
MRI findings. (a) The mass was visualized as low signal in T1WI. (b) The mass was visualized as high signal in T2WI. (c) On diffusion-weighted image, the mass was shown as high signal intensity. (d) On gadolinium ethoxybenzyl diethylenetriamine pentaacetic acid-enhanced MRI, the mass was shown as low signal intensity in hepatobiliary phase (yellow-dotted area).

She had been diagnosed with UC at 38 years old and prescribed 5-amynosalicylic acid until 49 years old. She had no drinking habit, but her mother had history of kidney cancer. Vital signs were within normal range. The abdominal pain remained resolved, and other abdominal findings were also normal. Laboratory tests, including aspartate aminotransferase, alanine transaminase, total bilirubin, albumin, alkaline phosphatase, *γ*-glutamyltransferase, platelet count, and prothrombin time, were within normal range. Tumor markers, such as *α*-fetoprotein, protein-induced vitamin K absence-II, carcinoembryonic antigen, and carbohydrate antigen 19-9, were also within normal range. Hepatitis B virus (HBV) surface antigen and hepatitis C virus (HCV) antibody were both negative. A low echoic mass (12.8 × 12.0 mm) with blood flow signal was detected in segment 3 of the liver on abdominal US (Figure 1). On plain CT, the mass was detected as a low-density lesion. The mass was not enhanced on contrast-enhanced CT in the arterial phase, was slightly enhanced in the portal phase, and was detected as low-density lesion in the delayed phase (Figures 2(a)–2(d)). On magnetic resonance imaging (MRI), the mass was recognized as low signal in T1-weighted image (T1WI) and high signal in T2-weighted image (T2WI). On diffusion-weighted image, the mass was shown as an area of high signal intensity. It was shown as an area of low signal intensity in the hepatobiliary phase on gadolinium ethoxybenzyl diethylenetriamine pentaacetic acid-enhanced MRI (Figures 3(a)–3(d)). Colonoscopy revealed the remission of UC, and there were no malignant findings on esophagogastroduodenoscopy. Definitive diagnosis was very difficult because there were no typical findings of malignant tumor, such as hepatocellular carcinoma or metastatic carcinoma. Furthermore, there was no elevation of tumor marker levels or hepatitis virus marker levels. As a differential diagnosis, highly differentiated hepatocellular carcinoma and slow-staining hemangioma were considered, but clear evidence was lacking. Regular image follow-up was recommended as watchful waiting, but the patient strongly requested surgery for diagnosis because of family history of malignant disease.

**Figure 4:**
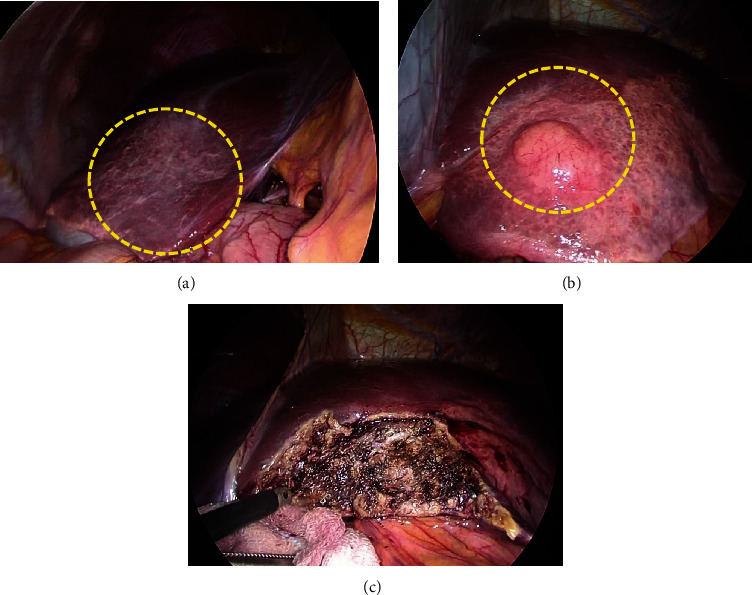
Intraoperative findings. (a) A discolored area was observed in the right lobe of the liver (yellow-dotted area), and it suggested chronic inflammation. (b) An area of the tone was also observed in left lobe, and a mass was observed in the area (yellow-dotted area). (c) Laparoscopic partial hepatectomy was performed.

**Figure 5:**
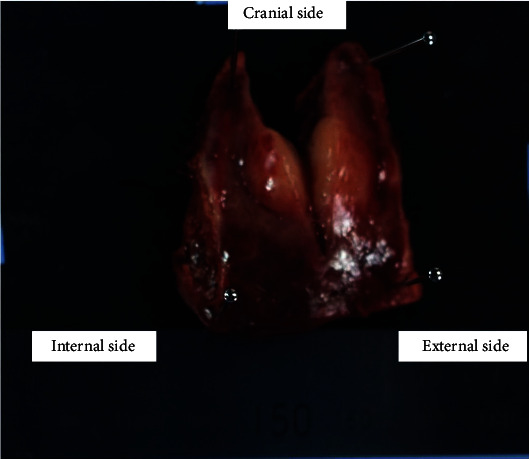
The resected specimen was a hard, elastic, well-demarcated whitish mass.

**Figure 6:**
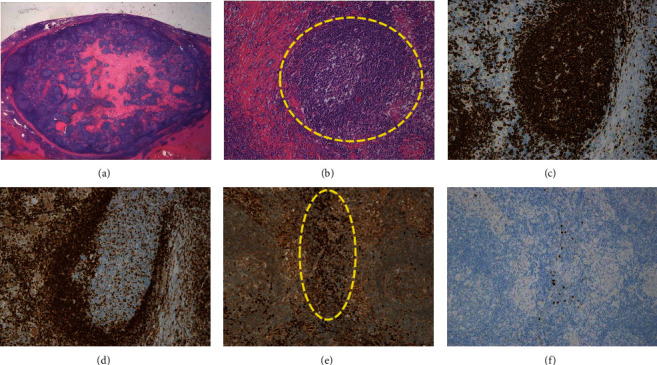
Histopathological findings. (a) HE low magnification. A geographical hyalinization was observed in the center of the lesion. (b) HE high magnification. It was a lesion showing confluent proliferation of lymphoid follicles with germinal center (yellow-dotted area). (c) Lymphoid follicles were CD20-positive. (d) Lymphoid follicles were Bcl-2-negative. (e) Mild IgG-positive histiocytes and plasma cells were observed between the follicles (yellow-dotted area). (f) There was no significant increase in IgG4-positive cells.

Surgery was planned, and laparoscopic partial hepatectomy was performed. In the operative findings, a discolored area was observed in both lobes of the liver, suggesting chronic inflammation. The mass was recognized at segment 3 in the discolored area (Figures 4(a)–4(c)). The resected specimen was an elastic, hard, well-demarcated whitish mass measuring 12 × 10 mm (Figure 5). Histopathological findings revealed a number of lymphoid follicles with a germinal center. Infiltrating lymphocytes were non-neoplastic and lymphoepithelial lesions were not recognized. On immunohistochemical staining, lymphoid follicles were CD20- and CD3-positive and Bcl-2-negative. There was no monoclonality on immunoglobulin (Ig) kappa and lambda. Mild IgG-positive histiocytes and plasma cells were observed between the follicles, but there was no significant increase in IgG4-positive cells (Figures 6(a)–6(f)). Inflammatory findings in the background liver tissue were mild. Pathological diagnosis was RLH of the liver. Postoperative course was uneventful, and the patient was discharged on postoperative day 6. Blood tests including anti-nuclear antibody (ANA), IgG, anti-Sm antibody, and anti-mitochondrial antibody were examined after the operation, but all were within normal range. The patient is currently under outpatient follow-up, but there have been no signs of recurrence after approximately two years. The patient also never developed recurrence of abdominal pain.

## 3. Discussion

RLH, also known as pseudolymphoma or nodular lymphoid lesion, is a benign disease characterized by the proliferation of non-neoplastic polyclonal lymphocytes that form follicles with reactive germinal centers; it rarely occurs in the liver [1, 2]. RLH predominately occurs in middle-aged women, with some type of autoimmune disease in about 40% of the cases [2]. Many patients have no symptoms, and they are often found incidentally on imaging study [2]. In our case, CT was performed to investigate the cause of abdominal pain happened to reveal a mass in the liver. However, we considered that there is no relationship between abdominal pain and liver mass because of its small size. Moreover, we have no explanation for the abdominal pain which was surely nonspecific.

There were no specific tumor markers for the RLH, but since the tumor was very small, so it is unlikely to be useful for diagnosis. The imaging study showed a small hypoechoic lesion on US, and hyper- to hypovascular on contrast CT or MRI was also nonspecific, so it was difficult to rule out hepatocellular carcinoma or metastatic carcinoma or even differentiate between each other [4–6]. In our case, the patient had no background of chronic hepatic inflammation such as HBV and HCV or notable use of alcohol. Additionally, the tumor markers were within all normal range. Therefore, she was firstly recommended watchful waiting follow-up because there were no typical findings of malignant disease. Surgery was performed owing to the strong demand of the patient, and as a result, it was considered appropriate because definitive diagnosis could be done and because of a previous report of a case in which lymphoma progressed after being diagnosed with RLH of the liver [7]. Preoperative diagnosis of RLH is thought to be difficult, and many cases have been diagnosed after surgical resection [2]. Preoperative liver biopsy may be useful for diagnosis, but several reports have pointed out the risk of dissemination if the lesion has the malignant potential [8, 9], so careful judgment is required. On pathological findings, RLH is also characterized by a localized, well-demarcated lesion, with the presence of hyperplastic lymphoid follicles with polyclonal and polymorphic small mature lymphocytes, macrophages, and plasma cells [10]. Diseases such as inflammatory myofibroblastic tumor, low-grade lymphomas with a nodular growth pattern, primary marginal zone lymphoma, and follicular lymphoma must be differentiated from RLH of the liver [2, 6]. Differentiation from lymphoma is especially very important. Reactive lymphoid follicles are also contained in marginal zone lymphoma, but lymphoepithelial lesions and cellular atypia were reported to be observed in mucosa-associated lymphoid tissue lymphoma, not in relation with RLH of the liver [6]. Additionally, follicular lymphoma often exhibits more densely packed follicles of uniform size and shape [6]. In our case, no atypical change was observed in the infiltrating lymphocyte, and there was no detection of monoclonality in Ig kappa or lambda. It suggested the non-neoplastic lesion. Lymphoepithelial lesions were not also observed, so mucosa-associated lymphoid tissue lymphoma was ruled out. Lymphoid follicles with germinal center were positive for CD-20 and negative for Bcl-2. Lymphoma, especially follicular lymphoma, therefore could be ruled out. Additionally, mild IgG-positive histiocytes and plasma cells were observed between the follicles, but there was no significant increase in IgG4-positive cells, so the IgG4 related diseases could also be ruled out. The pathogenesis of RLH of the liver is still unclear [1, 2, 4–6, 9]. Reactive immune phenomena to various immune stimulants are presumed to be the trigger, such as various autoimmune diseases, viral hepatitis, or cancer [1, 2]. Kanno et al. summarized 76 cases of RLH of the liver, 35.5% had liver disease, 17.1% had autoimmune disease, and 27.6% had malignant tumor [9]. Common diseases were reported to be viral such as hepatitis B, primary biliary cirrhosis (now called primary biliary cholangitis), autoimmune hepatitis, chronic thyroiditis, colon cancer, and gastric cancer [9]. In our case, HBV, HCV, ANA, IgG, anti-Sm antibody, and anti-mitochondrial antibody were all within normal range. Thyroid function test was not performed, but the patient had no signs of chronic thyroiditis such as fatigue, edema, or neck swelling. Furthermore, neither esophagogastroduodenoscopy nor colonoscopy revealed malignant findings, and no other malignant tumor was detected on CT or MRI. In the intraoperative findings, the discolored area observed in both lobes of the liver suggested some chronic inflammation. Our case had a history of UC, and UC is a chronic inflammatory bowel disease. Chronically inflamed and disturbed intestinal barrier in the UC may introduce a leakage of bacterial components into the portal circulation and subsequent portal inflammation [11]. Some inflammation may therefore be present in the liver of the patient with UC. A common liver lesion in UC is primary sclerosing cholangitis (PSC), and the prevalence was reported to be 2-8% [11, 12]. PSC is a chronic cholestatic liver disease of unknown origin and important hepatobiliary complication of UC [11], but our case did not reveal the histopathological findings of PSC. Additionally, localized lymphoid hyperplasia (LLH) consists a dense lymphoid infiltrate in the lamina propria and submucosa characterized by follicles with germinal centers, known to be associated with inflammatory disease and colonic carcinoma [13]. LLH of the rectum may be an early form of UC [13]. So, we considered the possibility that RLH of the liver is one of the hepatic lesions associated with UC. But the clear explanation about the relationship between the RLH and UC is difficult at this point, because UC was in remission by colonoscopy without taking medication and inflammatory findings in the background liver tissue were mild. Since Snover et al. firstly reported RLH of the liver [3], there have been only 79 cases reported in English [1–9, 14–16]. However, there have been no reports of cases with UC, and our case is thought to be the first. This case is therefore thought to be informative, and we believe that it will be helpful in the differential diagnosis of liver tumors in patients with UC in the future. In this case, hepatectomy was performed because of the strong demand of the patient. Surgery is the most appropriate option for making a definitive diagnosis, but it is important to take into consideration such as age, comorbidities, and surgical risks when making a final decision. For example, for an elderly patient or for a patient who has many comorbidities, needle biopsy and close radiological image follow-up may also be an option.

Another report states that malignant transformation of RLH in the liver is rare and diagnosis by biopsy is acceptable [6].

This time, we experienced a rare case of RLH of the liver with history of UC, but this is only a single case report and may have just occurred incidentally. Therefore, it is difficult to associate the two diseases. We suggest that there are likely to be cases that are not recognized as RLH and are overlooked, so it is necessary to accumulate cases.

## 4. Conclusions

To our knowledge, this is the first case of reactive lymphoid hyperplasia of the liver in a patient with history of ulcerative colitis that is reported. The association between both conditions however remains probably coincidental and speculative. Therefore, further investigation is necessary to accumulate reports.

## Data Availability

All data generated during this study are included in this published article.
